# Effect of denture wearing on occurrence of fungal Isolates in the oral cavity: A pilot study

**DOI:** 10.4317/jced.50656

**Published:** 2012-04-01

**Authors:** Shushant K. Garg, Varsha A. Singh, Sandeep K. Garg, Sanjeev Mittal, Gagandeep K. Chahal

**Affiliations:** 1 M.D.S. Professor & HOD. Deptt. Of Prosthodontics. M.M.College of Dental Sciences and Research, Mullana,Ambala; 2 M.D.S. Professor & HOD. Deptt. Of Microbiology. M.M.College of Medical Sciences and Research, Mullana,Ambala; 3 M.D.S. Professor. Deptt. Of Prosthodontics. M.M.College of Dental Science and Research, Mullana, Ambala; 4 M.D.S. PG Student. Deptt. Of Prosthodontics. M.M.College of Dental Sciences and Research, Mullana, Ambala

## Abstract

Objectives: An attempt was made to evaluate effect of denture wearing on occurrence of fungal isolates in the oral cavity before and after complete denture insertion. Method: Twenty five completely edentulous patients were selected; swab samples were collected intraorally before fabrication of complete dentures from labial vestibular area and after complete denture fabrication (one and four days after denture insertion). Further these samples were inoculated and incubated. Results: In nineteen patients no isolate of fungus before denture insertion as well as 4 days after denture insertion was found. In two subject results were false positive (contamination from environment), and in four patients there was increase in growth but not much significant increase of growth of fungal isolates was seen (mild growth of fungus only after denture insertion). One of the major finding of this study was overall occurrence of fungal isolates (before and after denture insertion) in the oral cavity were not significant.

** Key words:**Fungal isolates, denture stomatitis, denture, Candida, insertion.

## Introduction

The normal microbial flora of oral cavity is complex and consists of large number of species of bacteria including mycoplasma, fungi and protozoa. This is because of fact that mouth has many distinct habitats including saliva and crevicular fluids, surface of soft tissues such as lips, palate, cheek, tongue, gums and hard surfaces of teeth.

According, to several studies it has been stated that microbial flora varies qualitatively and quantitatively after tooth eruption, tooth extraction (i. e in edentulous patients), artificial denture, dental treatments (scaling, polishing, filling), the frequency and type of food ingested and antibiotic treatment. Healthy individuals usually exhibits many fungal species mainly Candida albicans in oral cavity which is a prevalent opportunistic pathogen in the oral cavity resulting in a multitude of fungal infection (1).

In denture wearers candidiasis is aggravated by the adhesion of Candida albicans to tissue-fitting surface of a maxillary denture base, which serves as an effective reservoir of micro-organisms. Large accumulations of hyphae and inflammatory cells have been found to present in denture wearers with denture stomatitis (2). Rare evidence was found regarding occurrence of all the fungal isolates after denture insertion.

So considering all these facts this pilot study was conducted to evaluate effect of denture wearing on occurrence of fungal isolates in the oral cavity.

## Material and methods

This was an in vivo study conducted in the Department of Prosthodontics including Crown and Bridge in M.M. College of Dental Sciences and Research, Mullana, Ambala. Twenty five patients were selected according to following criteria:

1) Completely edentulous patients who never wore complete denture before.

2) Patients were clinically healthy (not medically compromised).

3) Patients had good oral hygiene and clinically normal oral mucosa.

4) Patients were not on any kind of medication.

5) Patient were non allergic (had no past history of allergy to drugs, materials, food stuff etc).

6) Patient had no oral lesions when examined intraorally.

The study received approval from the ethical committee for the use of human subjects. All patients signed informed consent.

Collection of swab sample

Procedure of collection of swab sample for patient was carried out as follows:

Swab sample before denture insertion 

**Figure 1:**
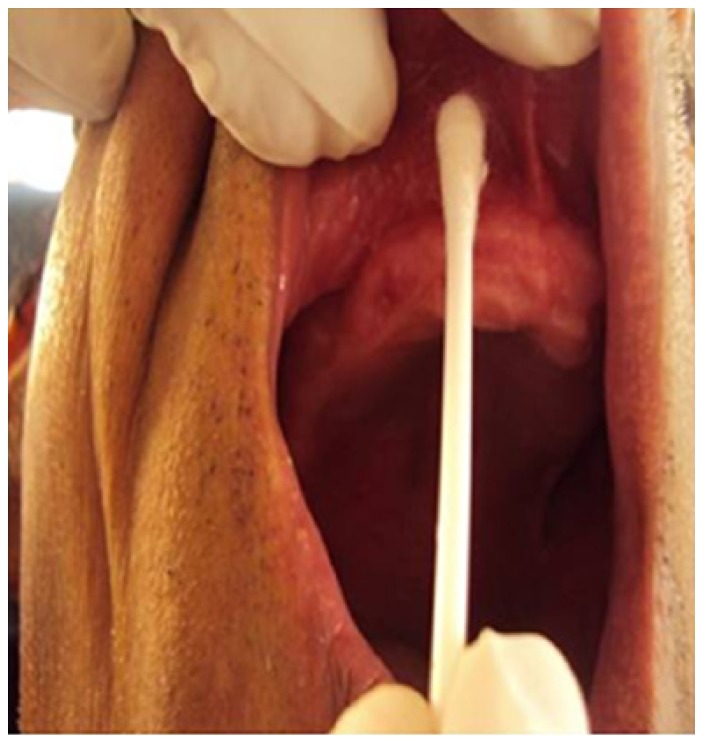
Swab sample was collected intraorally with sterile, culture device (HiMedia Laboratories Pvt limited, Mumbai, India) from maxillary labial vestibular area before complete denture insertion.

Swab sample was collected intraorally with sterile, culture device (HiMedia Laboratories Pvt limited, Mumbai, India) from maxillary labial vestibular area (Fig. 1) before complete denture insertion. As soon as sample was collected it was dipped in sterile, culture collecting device (HiMedia Laboratories Pvt limited, Mumbai, India), containing normal saline (Nirlife healthcare, Chennai, India) to prevent drying of swab sample.

Swab samples after denture insertion 

Two swab samples were collected intraorally from the maxillary labial vestibular area. One swab sample 1day after denture insertion and second swab sample four days after denture insertion. These samples were kept in Sterile, culture collecting device (HiMedia Laboratories Pvt limited Mumbai) containing normal saline to prevent drying of samples. After collection of samples microbial analysis were performed.

**Figure 2:**
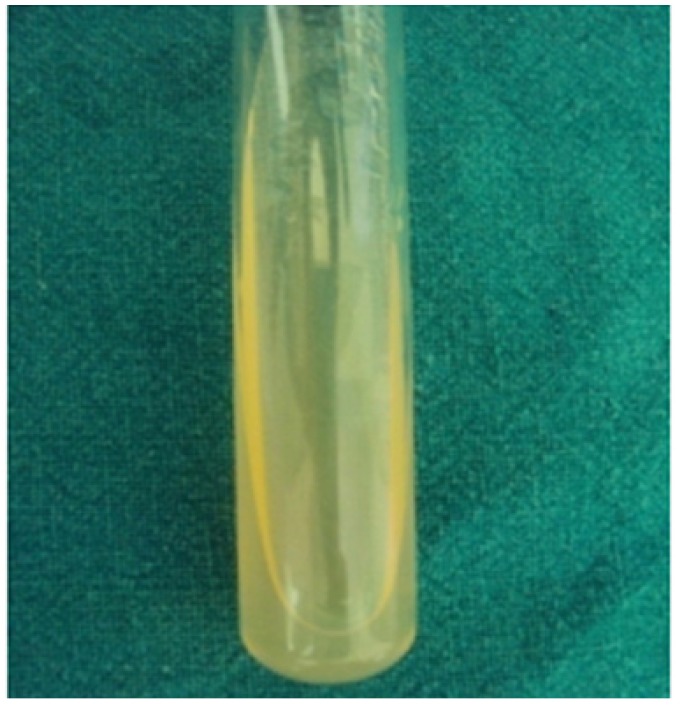
Culture media used for inoculation was Sabourauds Dextrose Agar (SDA) culture media (HiMedia Pvt limited, Mumbai, India) slant.

Microbial analysis of the samples: Swab samples from before denture and after denture insertion patient were inoculated on culture media with inoculating loops (Hitec Pvt limited Mumbai). Culture media used for inoculation was Sabourauds Dextrose Agar (SDA) culture media (HiMedia Pvt limited, Mumbai, India) slant (Fig. 2).

**Figure 3:**
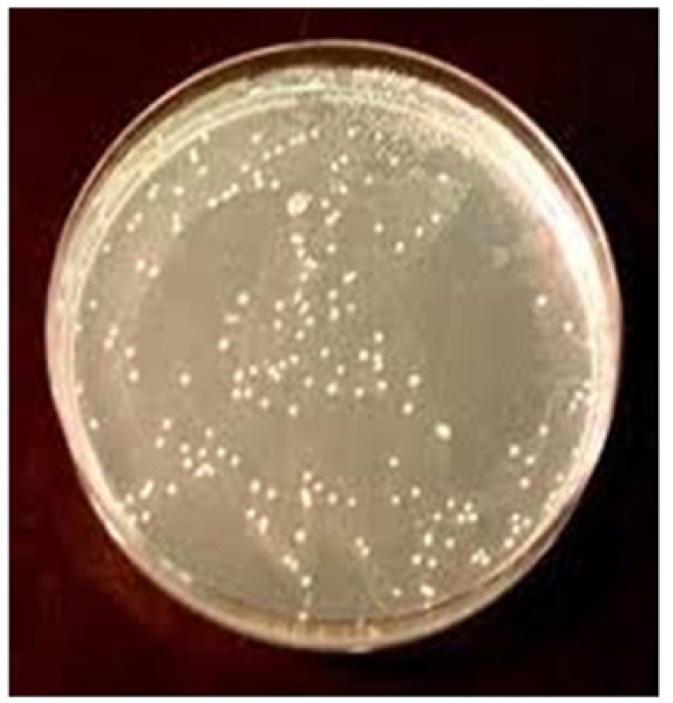
After proper incubation, growth or colonies were analyzed by gram staining and seen under microscope (Nikon, Eclipse E100) for microscopic analysis.

After inoculation with inoculating loops incubation was done at 37ºC for 48 hours in incubator (Hitec Pvt Ltd, Mumbai, India). After proper incubation, growth or colonies were analyzed by gram staining and seen under microscope (Nikon, Eclipse E100) for microscopic analysis. Further biometric analysis was carried out for confirmation of fungal growth and species detection. In some patients growth of fungal isolates was seen on SDA media (Fig. 3).

## Results

 Table 1 presents the presence of fungal growth before denture insertion .Only 1 patient showed positive growth for fungal isolate. Fungus isolated was of Candida species Candida albicans.

**Table 1:**
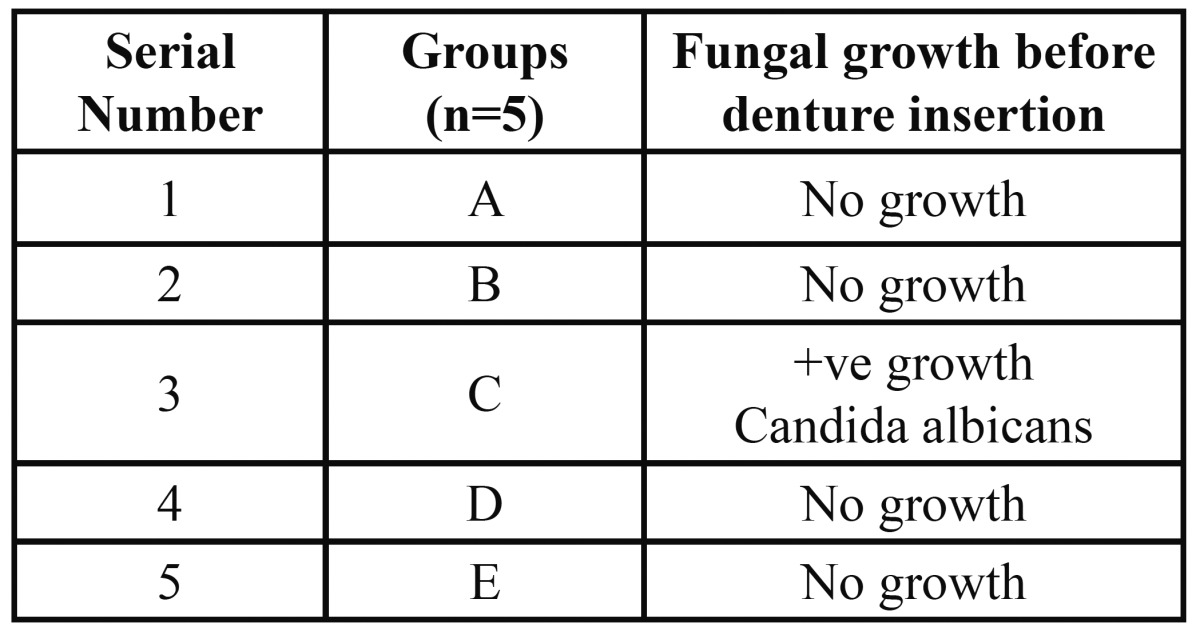
Fungal growth before denture insertion.

 Table 2 presents fungal growth 1 day after insertion of denture. 2 patients showed positive growth for fungal isolate. Fungus isolated was of Candida species Candida albicans.

**Table 2:**
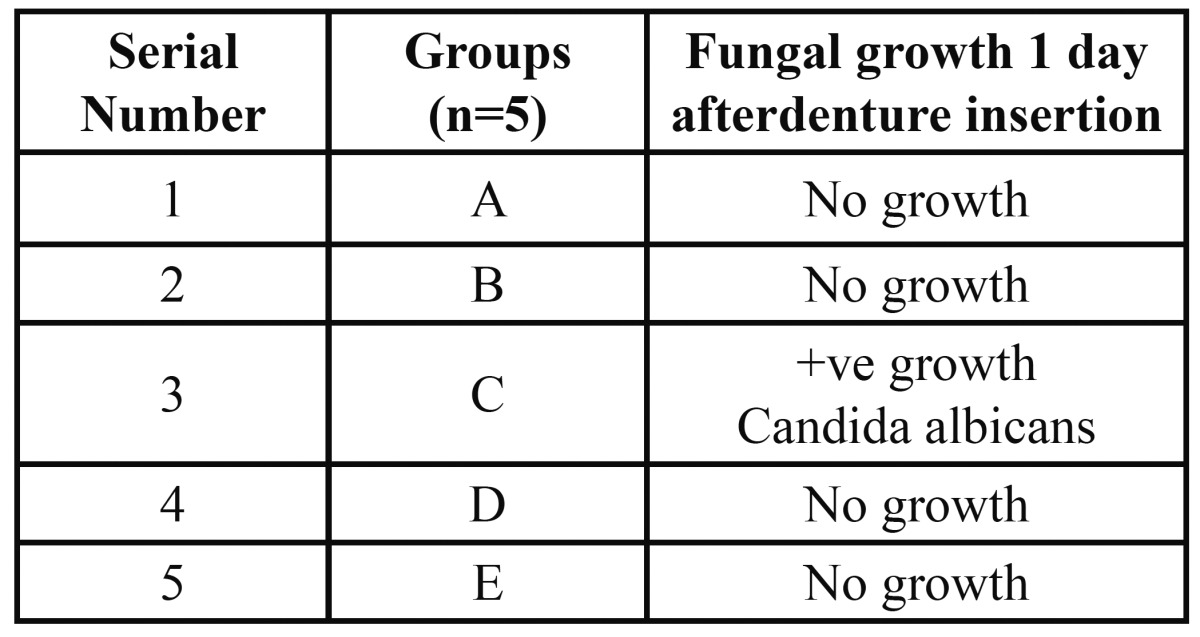
Fungal growth 1 day after denture insertion.

 Table 3 presents fungal growth 4 days after denture insertion 4 patients showed positive growth for fungal isolate. Fungus isolated was of Candida species Candida albicans.

**Table 3:**
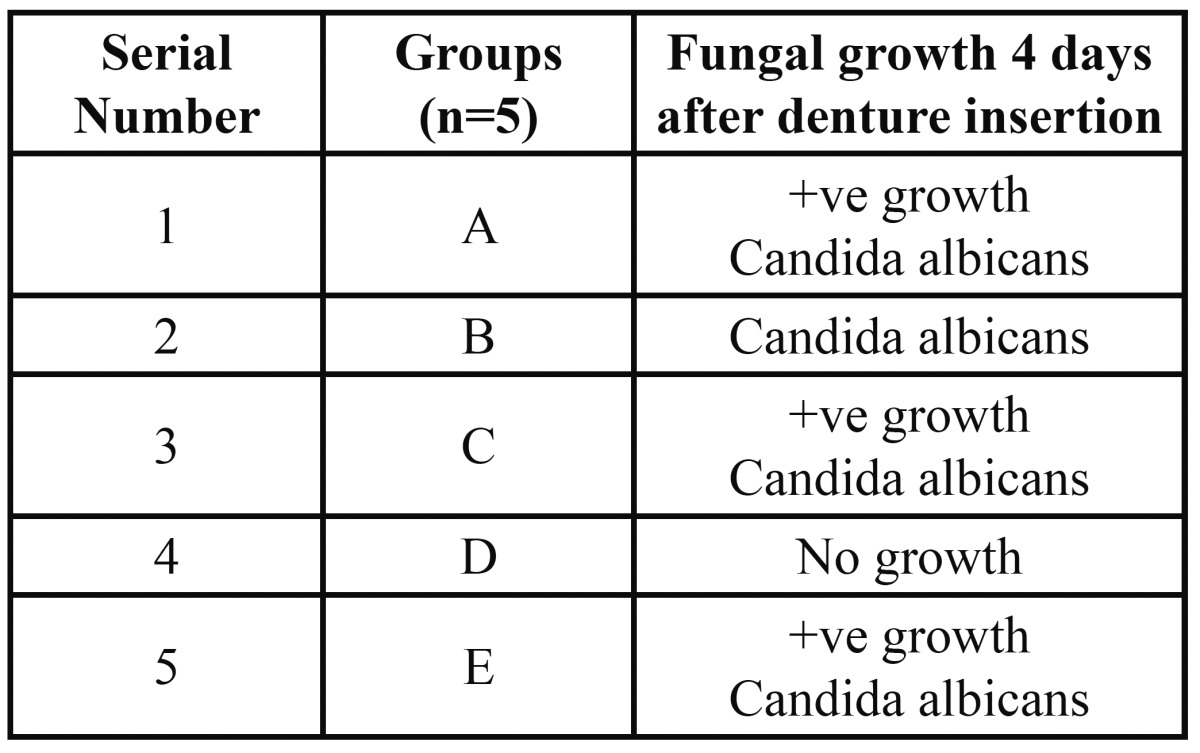
Fungal growth 4 day after denture insertion.

 Table 4 presents comparison of fungal growth before and after denture insertion.

**Table 4:**
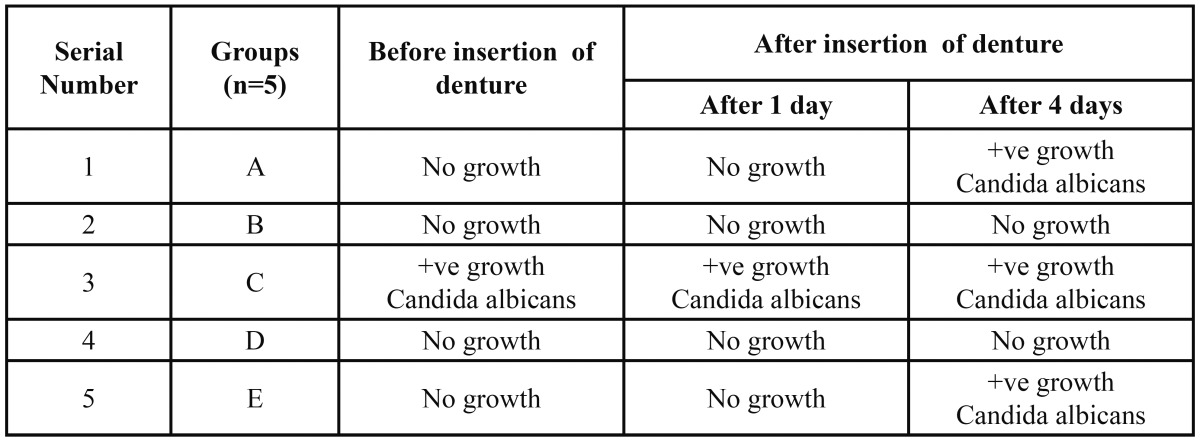
Comparison of fungal growth before and after denture insertion.

Chi square test:

The chi-Square test can be used to calculate the p-value by comparing the value of the statistic to a chi-square distribution .The number of degrees of freedom is equal to the number of possible outcomes, minus1.

Before insertion of denture chi-sq= 2.14,. d.f =4; p= 0.71which.

After one- day of insertion of denture chi-sq= 4.62, d.f = 4; p= 0.386 

After 4th - day of insertion of denture chi-sq= 2.5, d.f = 4; p= 0.3536 

P- Values and chi-sq test values presented that results were not –significant i.e. there was no significant effect of denture insertion on occurrence of fungal isolates in oral cavity after 1 and 4 days.

## Discussion

Fungi are the normal residents of the human body flora and most of them are potential pathogens. Fungal infections are called opportunistic because the fungi exploit a situation that is opportune for them, namely weakness of the host. Along with bacteria, fungai (yeasts) are the principal factors in the initiation, aggravation, and maintenance of a denture stomatitis (3,4,5).

Candida albicans is a prevalent, opportunistic fungal pathogen in the oral cavity resulting in a multitude of Candidal infections (6). The involvement of Candida as a potential causative agent in denture induced stomatitis was first described by Cahn in 1936. Candida albicans remains the most frequently isolated fungai in the oral cavity but other species have also been isolated and involved in disease.

In denture wearers, Candidiasis is aggravated by the adhesion of Candida albicans to the tissue surface of the maxillary denture base, which serves as an effective reservoir of microorganisms. Predisposing systemic and local factors induce the transformation of this commensal organism to a pathogen.

In vitro adherence studies illustrate that Candida albicans attaches readily to various resins, glass, and metal surfaces. The ability of Candida albicans to adhere to polymeric surfaces has been correlated with attractive hydrophobic and repulsive electrostatic forces. Surface characteristics resulting from chemistry are significant in the initial adherence of Candida albicans to the denture resin and offer an opportunity for further bonding and colonization (7,8) .Hence the present study was done to know the occurrence of fungal isolates before and after wearing of complete denture.

In the present study the data collected was based on the observation of fungal growth, before and after insertion of complete denture in the labial vestibular area before and after insertion of complete denture patients at varying time periods viz. before insertion of complete denture, 1day and 4 days after denture insertion.

Results showed that there was not much significant evidence of fungal growth before complete denture insertion and on the 1st day of insertion of complete denture insertion. This evidence may be as a result of proper oral hygiene of patient as well as good health of patient (9-13). One of the factors that may lead to less significant growth of fungal isolates was that study was carried out for short duration of time i.e after 1 day as well as after 4 days, increase in time duration might lead to occurrence of fungal isolates in increased number of patients. All these conditions attributed to the failure of isolation of fungal growth as observed in this study.

According to studies it has been seen that Denture stomatitis is frequent among denture wearers and varies widely; reported prevalence ranges from 10% to 75% (14).The etiology appears to be multiparametric; old age and concomitant decline of the immune defenses, systemic diseases, smoking, wearing dentures at night and poor oral hygiene resulting in the accumulation of plaque on the dentures have all been proposed as predisposing factors (15).

Increased susceptibility to general and oral superficial infection with yeasts has been associated with diabetic condition (16,17). Bahn and others have reported that the yeast, Candida albicans, was isolated in greater quantities from saliva of patients with diagnosed diabetes mellitus than from patients without diagnosis of diabetes (18).

Less than 20% of results showed there was isolation of fungal isolates (4 patients out of 25) but growth of fungus isolated was not much significant which was of prime concern in this study. Moreover 2 results in this study were false positive that means there was contamination.

Contamination may be:

A. At the time of collection of sample.

B. At the time of processing of sample.

At the time of collecting of sample if the swab collecting instrument was not properly sterilized or if after collecting swab sample was exposed to environment for longer duration of time there can be false positive results. Other possible reason can be contamination of media at the time of preparation in the lab in which the swab sample will be inoculated, during inoculation if inoculating loops were not properly sterilized can lead to contamination.

From literature various factors may contribute to increase or decrease in fungal growth these may include oral hygiene of patient, immune status of patient and the medication patient is taking. So, all these factors were considered in this study.

Limitations of this study were smaller sample size, intraoral samples were collected only from maxillary labial vestibule area, the patients were followed for maximum duration of 4 days that can be increased for further evaluation.

Before denture insertion occurrence of fungal isolates was not significant i.e out of 25 patients only 1 patient showed positive growth.

After 1 day of denture insertion only 2 patients showed positive growth for occurrence of fungal isolates which was not significant. Again after 4 days when occurrence of fungal isolates was evaluated it was found that out of 25 patients in 4 patients positive growth of fungal isolates was depicted, though number of patients increased for occurrence of fungal isolates after 4 days of denture insertion but it was not significant result.

## Conclusion

Before denture insertion occurrence of fungal isolates was not significant i.e out of 25 patients only 1 patient showed positive growth.

After 1 day of denture insertion only 2 patients showed positive growth for occurrence of fungal isolates which was not significant. Again after 4 days when occurrence of fungal isolates was evaluated it was found that out of 25 patients in 4 patients positive growth of fungal isolates was depicted, though number of patients increased for occurrence of fungal isolates after 4 days of denture insertion but it was not significant result.
